# Supplementary material for: Structure of the human heparan sulfate polymerase complex EXT1-EXT2
Source: Nat Commun. 2022 Nov 19;13:7110. doi: 10.1038/s41467-022-34882-6 (PMC9675754; doi:10.1038/s41467-022-34882-6)
Supplement: Supplementary file 1 — Supplementary Information [file 41467_2022_34882_MOESM1_ESM.pdf]

Supplementary Information for

Structure of the human heparan sulfate polymerase complex EXT1-EXT2

Francisco Leisico[1,$], Juneina Omeiri[1,$], Christine Le Narvor[2], Joël Beaudouin[1], Michael Hons[3], Daphna Fenel[1], Guy Schoehn[1], Yohann Couté[4], David Bonnaffé[2], Rabia Sadir[1], Hugues Lortat-Jacob[1,*], Rebekka Wild[1,*]

[$]These authors contributed equally to this work
*Correspondence: Hugues.Lortat-Jacob@ibs.fr; Rebekka.Wild@ibs.fr

Supplementary Figures

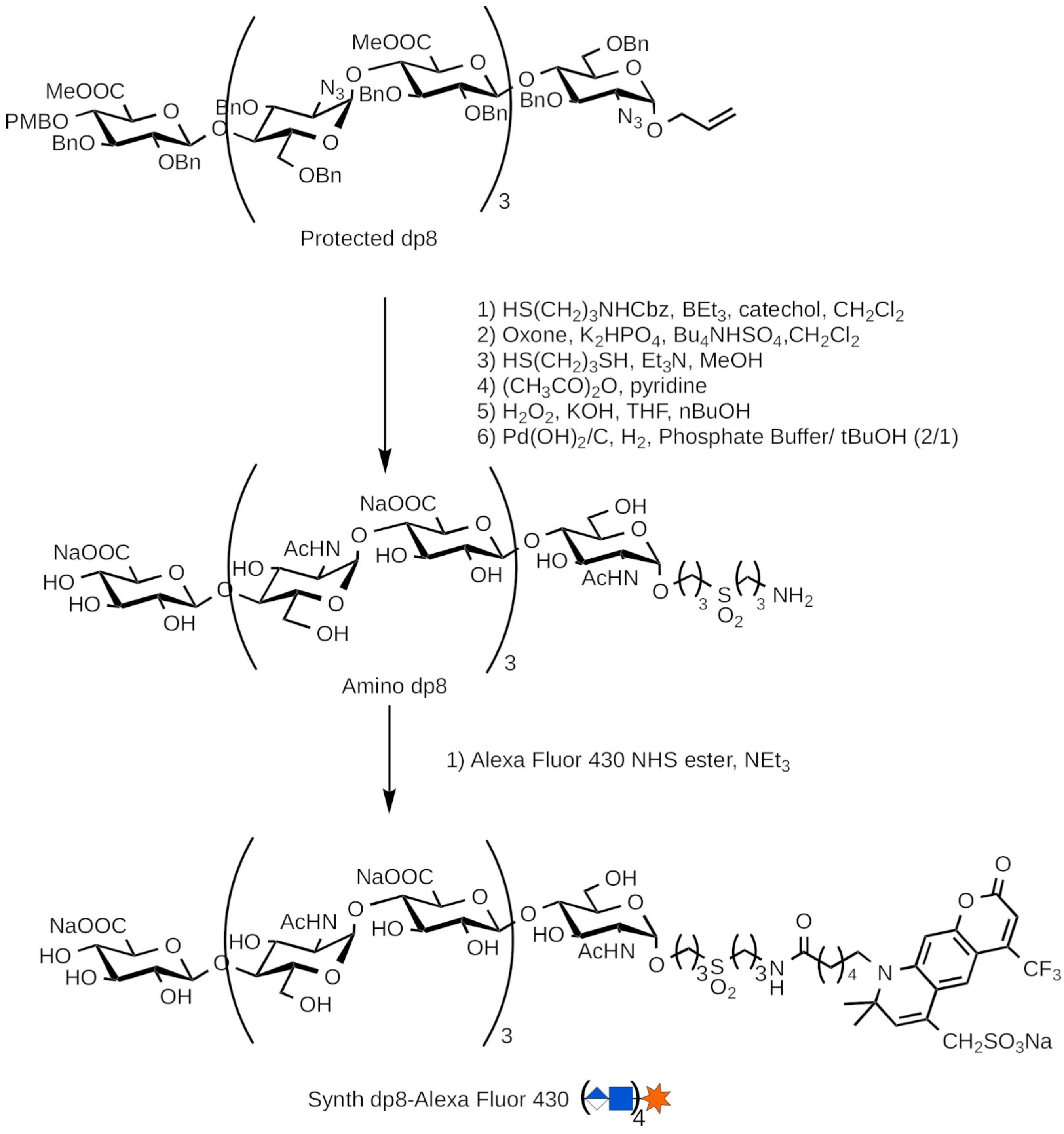

Supplementary Fig. 1 | Preparation of the octa-saccharide substrate analog. Seven step chemical synthesis of synthetic dp8-Alexa Fluor 430, an octa-saccharide consisting of repeating GlcNAc and GlcA units with a Alexa-fluorophore at the reducing end, from a protected dp8 precursor.

a

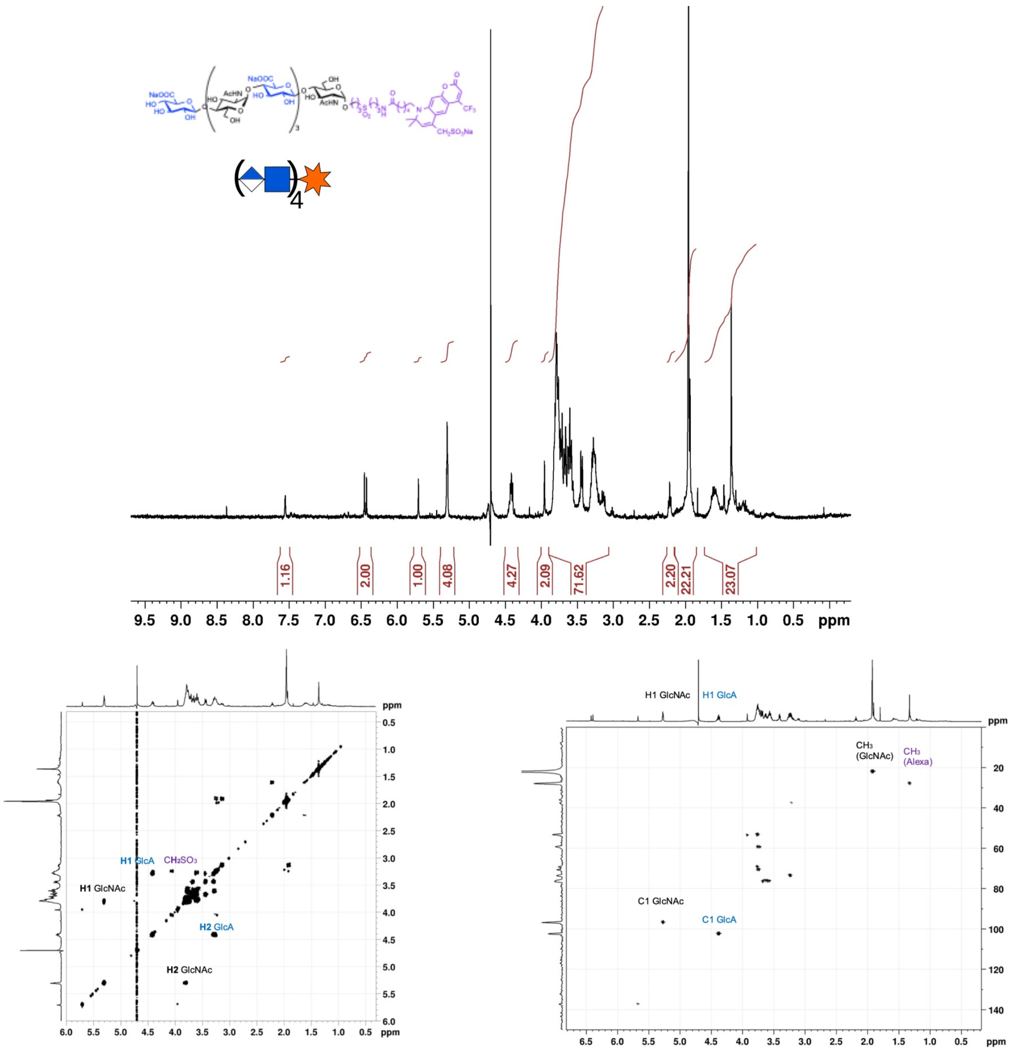

b

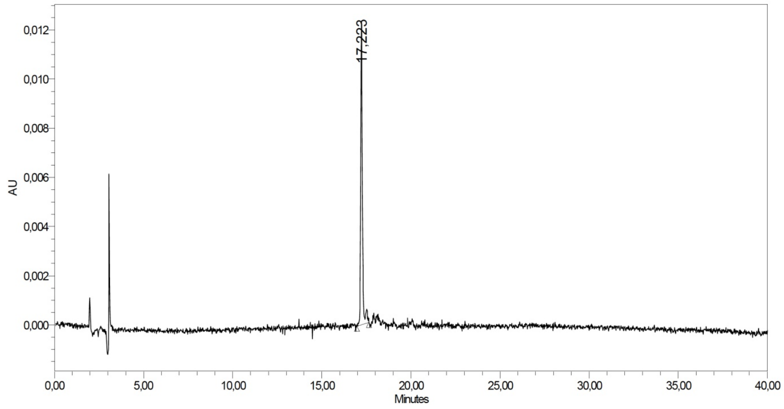

Supplementary Fig. 2 | Characterization of the octa-saccharide substrate analog. a NMR (1H, COSY, HSQC) spectroscopy of the synth dp8-Alexa Fluor 430. NMR analysis reveals expected signals at characteristic ^{1}H and ^{13}C chemical shifts of GlcNAc: H1/C1 (5.27/97.4 ppm), H2/C2 (3.77/69.2 ppm), CH_3 (1.93/21.8 ppm); GlcA: H1/C1 (4.39/103.1 ppm), H2/C2 (3.25/73.7 ppm); and Alexa Fluor 430: CH_2SO_3Na (3.92/53.8 ppm), CH_3 (1.32/28.6 ppm). ^{13}C chemical shifts were derived from ^{1}H-^{13}C HSQC experiment. **b** HPLC chromatogram of synth dp8-Alexa Fluor 430 indicating 95% purity based on absorbance at 430 nm.

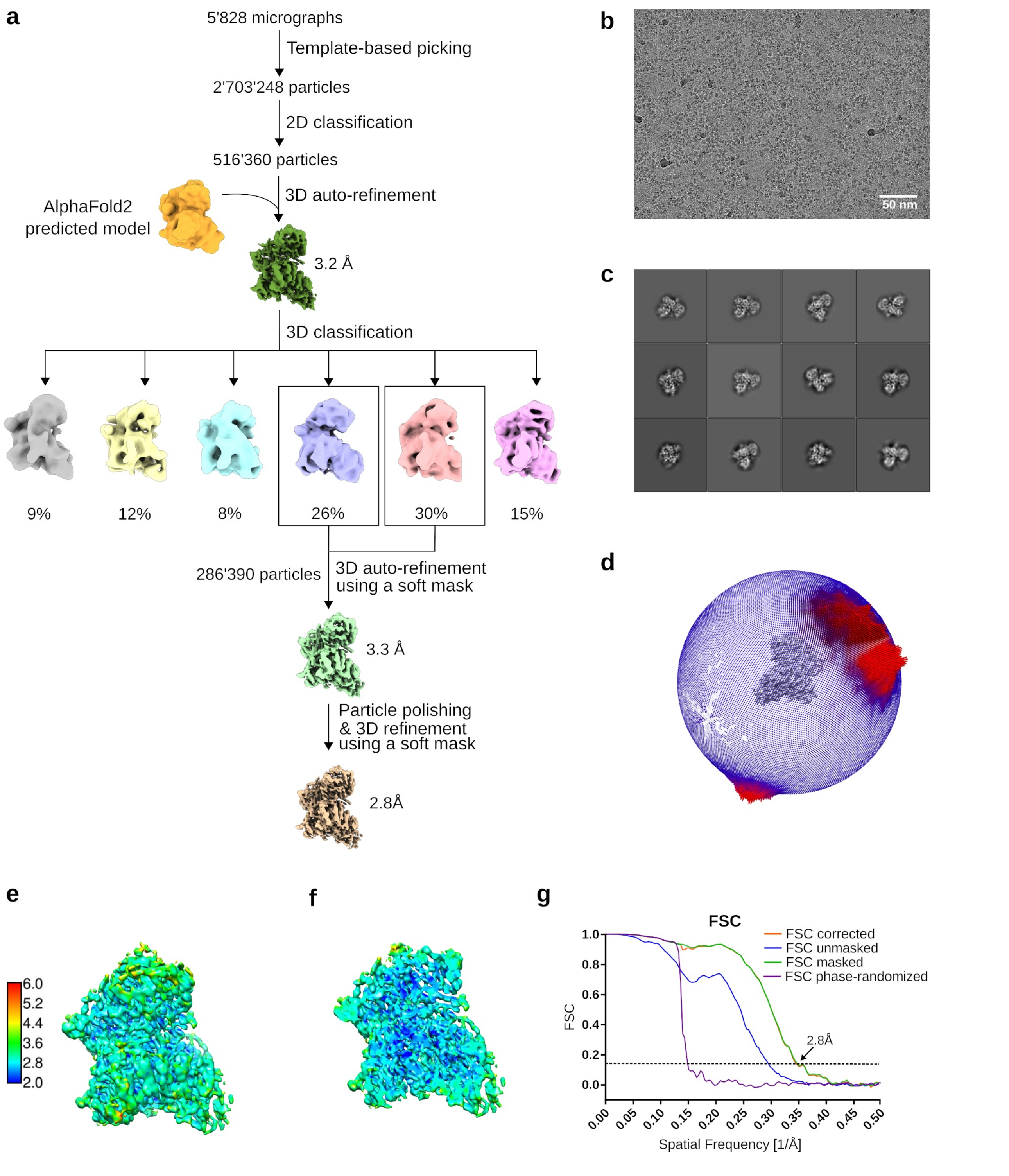

Supplementary Fig. 3 | EM data processing and quality assessment for human EXT1-EXT2. a Flow-chart of cryo-EM data processing procedure using RELION-3.1. **b** Representative motion-corrected and dose-weighted micrograph. **c** Selected class averages from 2D classification. **d** Angular distribution of particles in the final iteration of 3D refinement. **e** B-factor sharpened EM map, colored by local resolution as estimated in ResMap. **f** Map and representation as in (e) but vertically sliced. **g** Fourier shell correlation (FSC) curve indicating estimated resolutions based on the FSC = 0.143 criterion as generated by RELION-3.1.

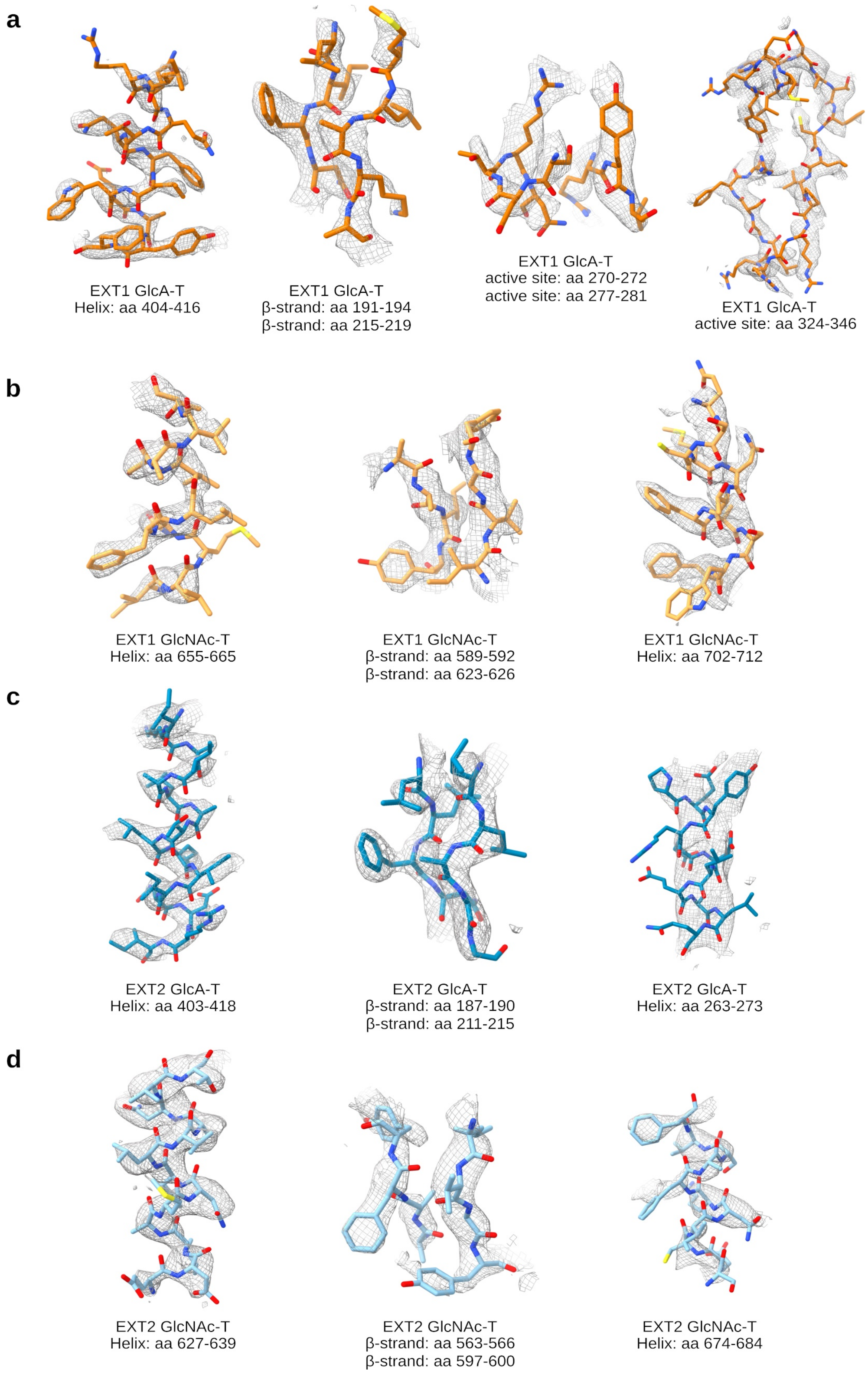

Supplementary Fig. 4 | Quality of EM map in different regions of the EXT1-EXT2 structure. **a** EXT1 GlcA-T; **b** EXT1 GlcNAc-T; **c** EXT2 GlcA-T and **d** EXT2 GlcNAc-T. Exemplary regions with varying map quality were chosen for illustration. GlcNAc-T, *N*-Acetylglucosamine-transferase; GlcA-T, glucuronic acid-transferase.

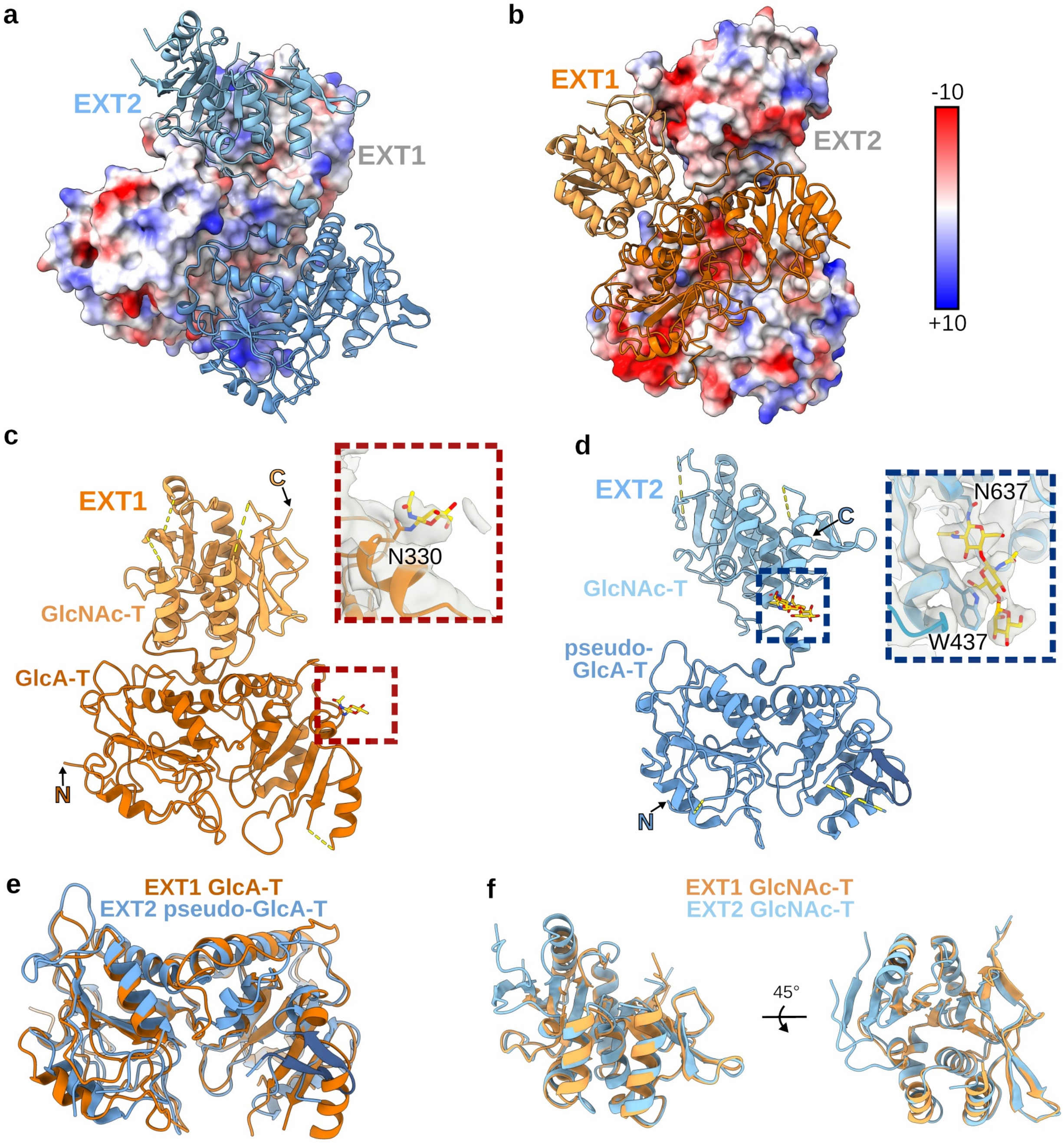

Supplementary Fig. 5 | EXT1 and EXT2 structure comparison. a Surface of EXT1 is colored according to its electrostatic potential with isocontours from blue (+10 κT/e) to red (-10 κT/e). EXT2 is shown in ribbon representation with its pseudo-GlcA-T and GlcNAc-T domain colored in dark and light blue, respectively. **b** EXT2 is shown in surface representation and colored according to its electrostatic potential. EXT1 is shown in ribbon representation with its GlcA-T and GlcNAc-T domain colored in dark and light orange, respectively. **c** Cartoon representation of EXT1. Close-up view shows a *N*-glycan (GlcNAc) in yellow stick representation and surrounding EM map in gray. **d** Cartoon representation of EXT2. Close-up view shows a well-ordered *N*-glycan (GlcNAc-GlcNAc-mannose), forming a Pi-stacking interaction with Trp437. **e** Superposition of EXT1 and EXT2 GlcA-T domains with a resulting R.M.S.D of 1.2 Å for 209 of 291 residues. **f** Superposition of EXT1 and EXT2 GlcNAc-T domains shown from two orientations. R.M.S.D is 0.96 Å for 150 out of 182 aligned residues. GlcNAc-T, *N*-Acetylglucosamine-transferase; GlcA-T, glucuronic acid-transferase.

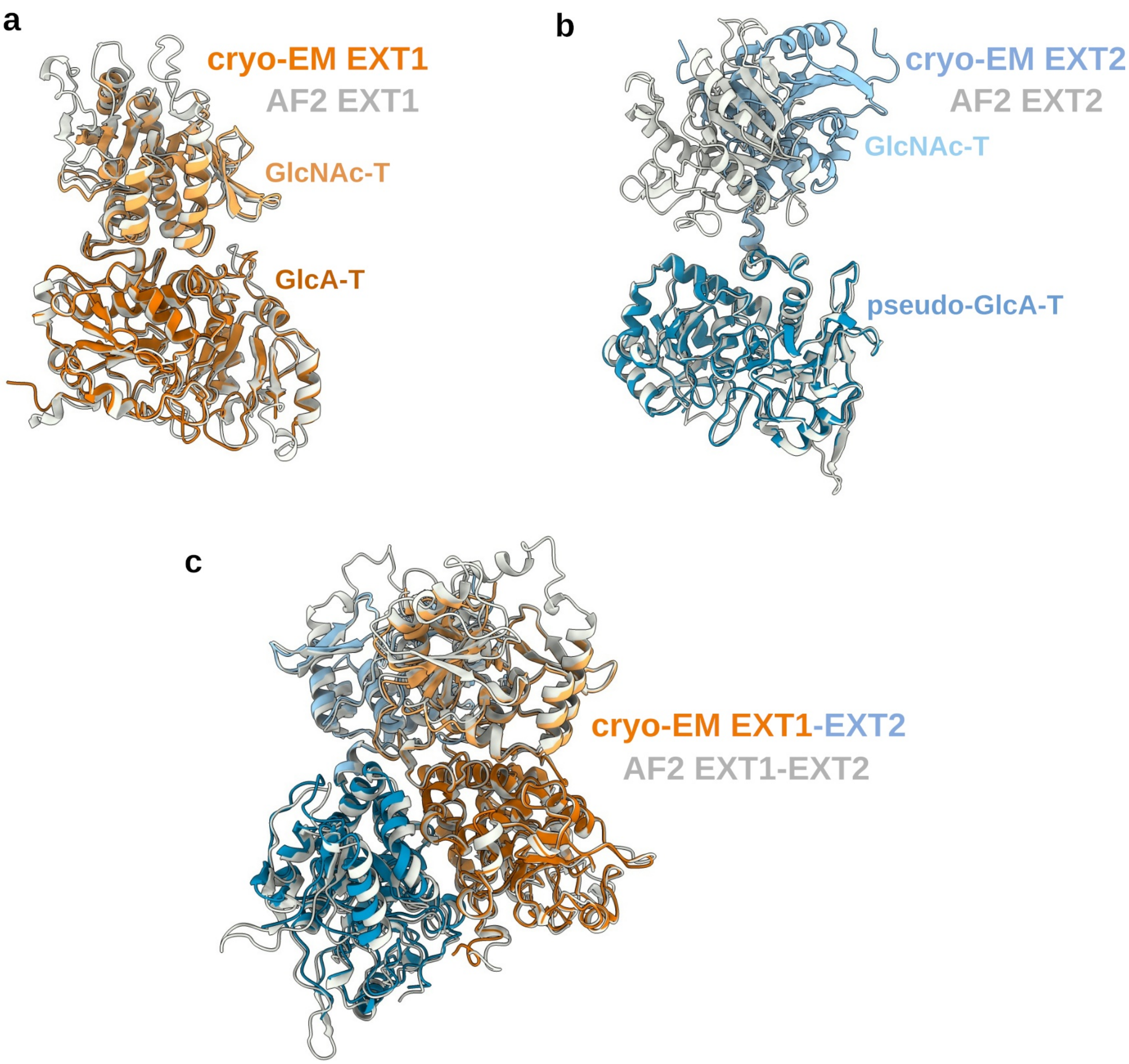

Supplementary Fig. 6 | Comparison of the EXT1-EXT2 cryo-EM structure with AlphaFold2 predicted models. a Superposition of EXT1 protein from the cryo-EM complex structure (in orange) and its predicted model (UniProt Q16394, in grey) using AlphaFold2[1]. **b** Cartoon representation of the EXT2 cryo-EM structure (in blue) and the AlphaFold2 predicted model (UniProt Q93063, in grey). Structures were aligned on the pseudo-GlcA-T domain. **c** Structural comparison of the EXT1-EXT2 cryo-EM complex structure with a predicted model for the hetero-dimeric complex using ColabFold[2]. GlcNAc-T, *N*-Acetylglucosamine-transferase; GlcA-T, glucuronic acid-transferase.

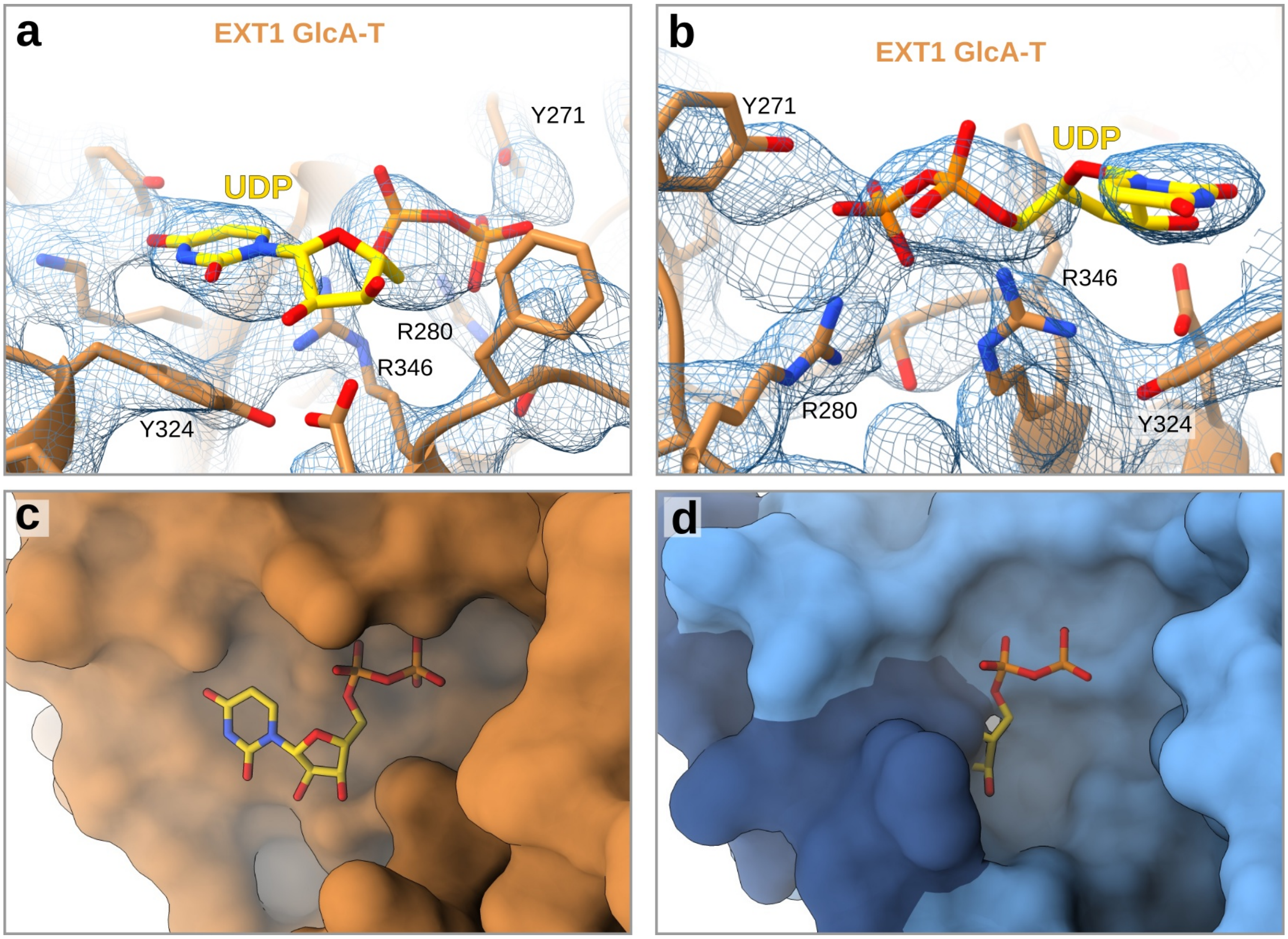

Supplementary Fig. 7 | UDP ligand binding in the EXT1 GlcA-T active site. a The EXT1 glucuronic acid-transferase (GlcA-T) active site is shown in ribbon and stick representation and colored in orange. The UDP ligand is shown as yellow sticks and the EM map is shown as blue mesh at a contour level of 0.02129. **b** Rotated view of EXT1 GlcA-T active site. **c** Surface representation of the UDP binding pocket in EXT1 colored orange with UDP ligand shown as yellow sticks. **d** The EXT2 pseudo-GlcA-T site is shown from the same view and colored in blue. EXT1 and EXT2 were superimposed to illustrate the potential UDP binding site in EXT2. The pocket is occupied by an anti-parallel beta-sheet (surface colored dark blue).

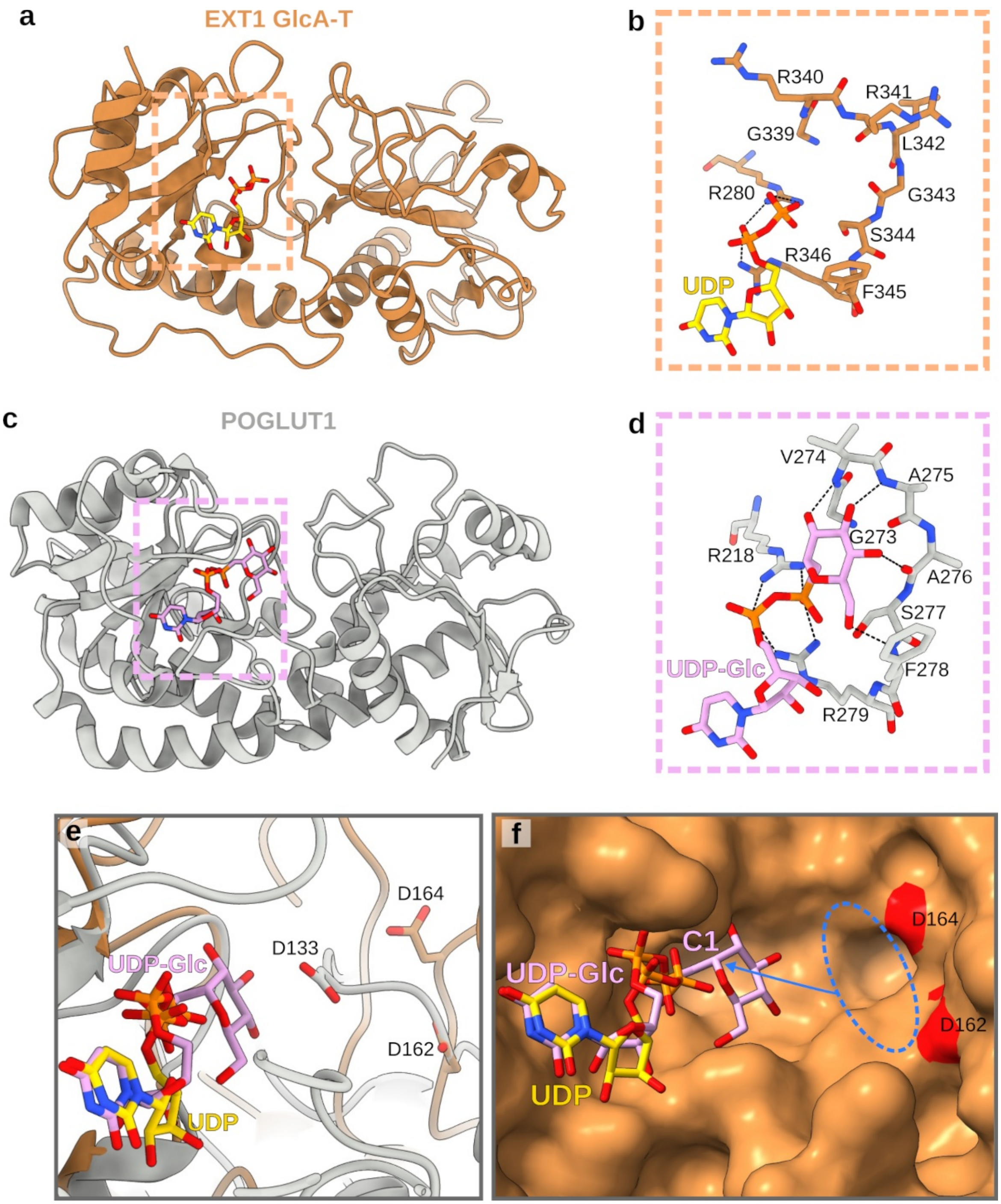

Supplementary Fig. 8 | Substrate binding by EXT1 GlcA-T and human POGLUT1. a Cartoon representation of the EXT1 glucuronic acid-transferase (GlcA-T) domain shown in dark orange with bound UDP ligand shown as yellow sticks. **b** Close-up view onto the potential UDP-GlcA substrate recognition loop shown in stick representation. Two critical arginines (R280 and R346) are also shown. Distances indicated as dotted lines are between 2.7 and 3.2Å. **c**, Crystal structure of human POGLUT1 (PDB-ID: 5L0U), a protein *O*-glucosyltransferase, shown in cartoon representation and colored in gray. The bound UDP-glucose (Glc) substrate analog is shown in stick representation and colored in plumb. **d** Close-up view shows hydrogen bond network between the protein backbone and the glucose, and between arginine residues (R218 and R279) and the

two phosphates. The G273-F278 loop accommodating the Glc moiety in POGLUT1 is similar to the loop G339-F345 in the EXT1 GlcA-T domain. Dotted lines in the close-up view indicate interactions with a distance between 2.9 and 4.0Å. **e** Superposition of the active sites of EXT1 GlcA-T (in orange) and POGLUT1 (in grey) shown as ribbon, with residues potentially acting as base catalysts shown as sticks. The UDP and UDP-Glc donor substrates are also shown as sticks. **f** View onto the catalytic site of EXT1 GlcA-T displayed in surface representation with bound UDP ligand shown as yellow sticks. Two aspartate residues potentially involved in acceptor substrate recognition are colored in red. Human POGLUT1 and EXT1 GlcA-T were superimposed and UDP-Glc ligand of POGLUT1 is shown as plum-colored sticks. The potential binding site of the oligosaccharide acceptor substrate is indicated with a blue ellipse and an arrow shows the direction of attack on the C1-carbon, characteristic for a S_N2-like reaction mechanism.

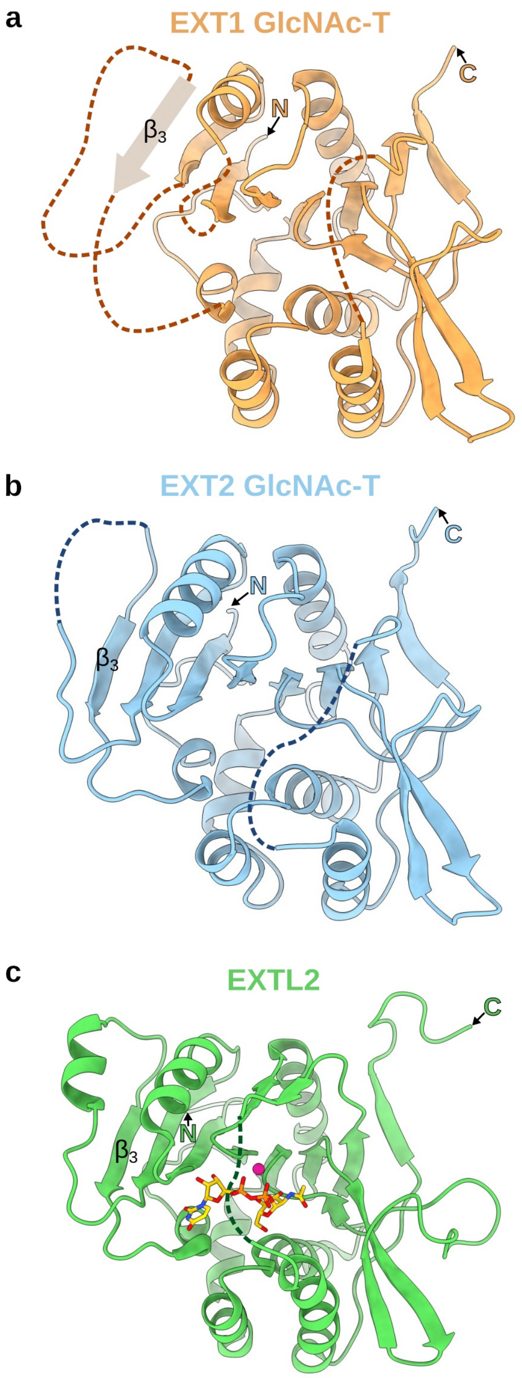

Supplementary Fig. 9 | The architecture of the EXT1 and EXT2 GlcNAc-T domains is highly similar to EXTL2. a Cartoon representation of the EXT1 *N*-Acetylglucosamine-transferase (GlcNAc-T) domain colored in orange. The missing beta-strand β_3 was drawn by hand. **b** EXT2 GlcNAc-T domain is shown as cartoon representation and colored blue. **c** Cartoon representation of the crystal structure of EXTL2 (PDB-ID: 1ON6) colored in green with bound UDP-GlcNAc substrate shown as yellow sticks and manganese as pink sphere. Missing loops are indicated as dotted lines.

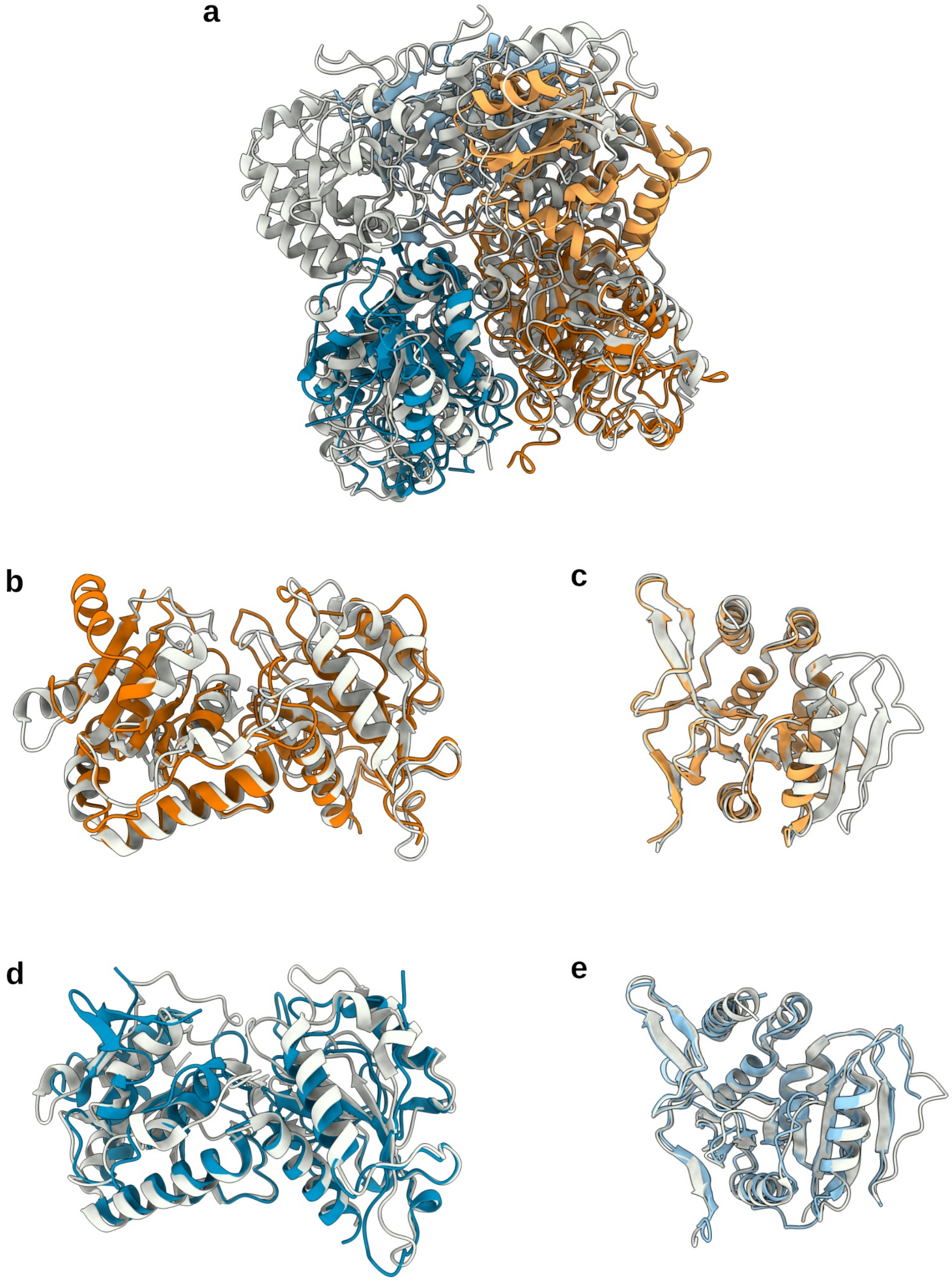

Supplementary Fig. 10 | Comparison of the hetero-dimeric EXT1-EXT2 and homo-dimeric EXTL3 complex structures. a Superposition of EXT1-EXT2, colored in blue and orange, with EXTL3, shown in gray (PDB-ID: 7AUA)[3]. Structures were aligned on the EXT1 and EXTL3 GlcA-T domains. In contrast to EXTL3, EXT1-EXT2 does not have C_2 symmetry, resulting in a distinct interdomain organization and a different overall architecture of the complex. **b** Superposition of EXT1 and EXTL3 GlcA-T domains with a resulting R.M.S.D. of 1.1 Å for 143 out of 301 residues. **c** Superposition of EXT1 and EXTL3 GlcNAc-T domains with a resulting R.M.S.D. of 0.8 Å for 160 out of 184 residues. **d** Superposition of EXT2 pseudo-GlcA-T and EXTL3 GlcA-T domains with a resulting R.M.S.D. of 1.1 Å for 140 out of 311 residues. **e** Superposition of EXT2 and EXTL3 GlcNAc-T domains with a resulting R.M.S.D. of 1.0 Å for 198 out of 216 residues. GlcNAc-T, *N*-Acetylglucosamine-transferase; GlcA-T, glucuronic acid-transferase.

a

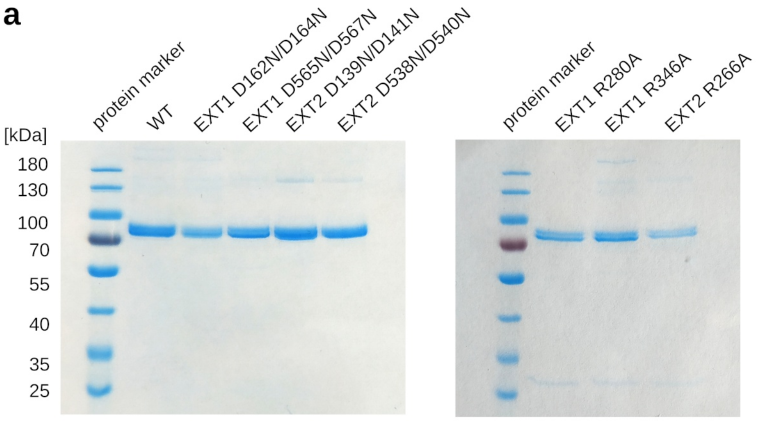

b

Sample	TM1 [°C]	TM2 [°C]
WT	54.2	54.3
EXT1 D162N/D164N	54.1	54.3
EXT1 D565N/D567N	47.9	47.8
EXT2 D538N/D540N	52.6	52.6
EXT1 R280A	54.9	54.9
EXT1 R346A	51.4	51.5
EXT2 R266A	52.8	54.0

c

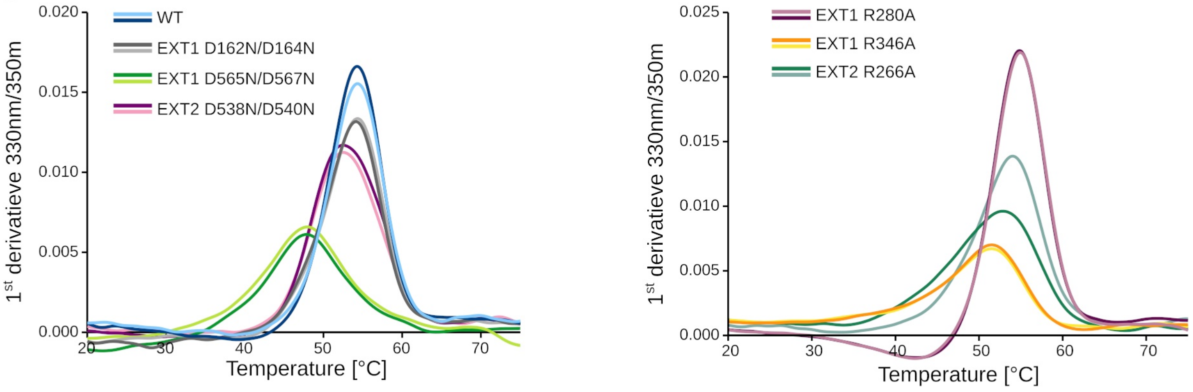

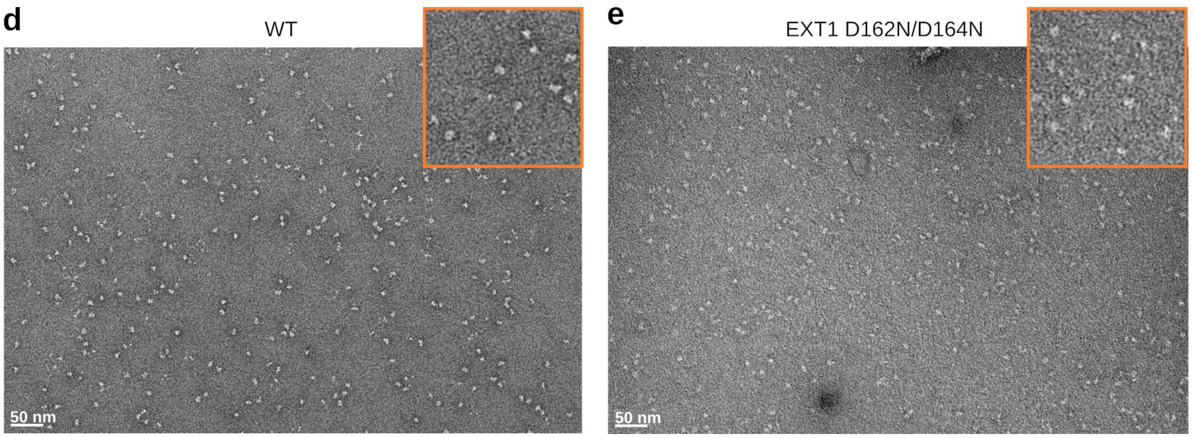

Supplementary Fig. 11 | Quality and stability assessment of purified EXT1-EXT2 complexes harboring point mutations. a SDS-PAGE analysis of purified EXT1-EXT2 wild-type (WT) and mutant complexes. Source data is provided as a Source Data 8. **b** Table summarizing the melting temperatures observed in thermal stability measurements of purified EXT1-EXT2 WT and mutant complexes using nano differential scanning fluorimetry. Measurements were performed in duplicate. **c** Melting curves of thermal stability measurements described in (b). Source data is provided as a Source Data 9. **d-e** Negative stain EM analysis of the wild-type and EXT1 D162N/D164N containing EXT1-EXT2 complex. Zoom-in into an area of the micrograph is shown in the top right corner.

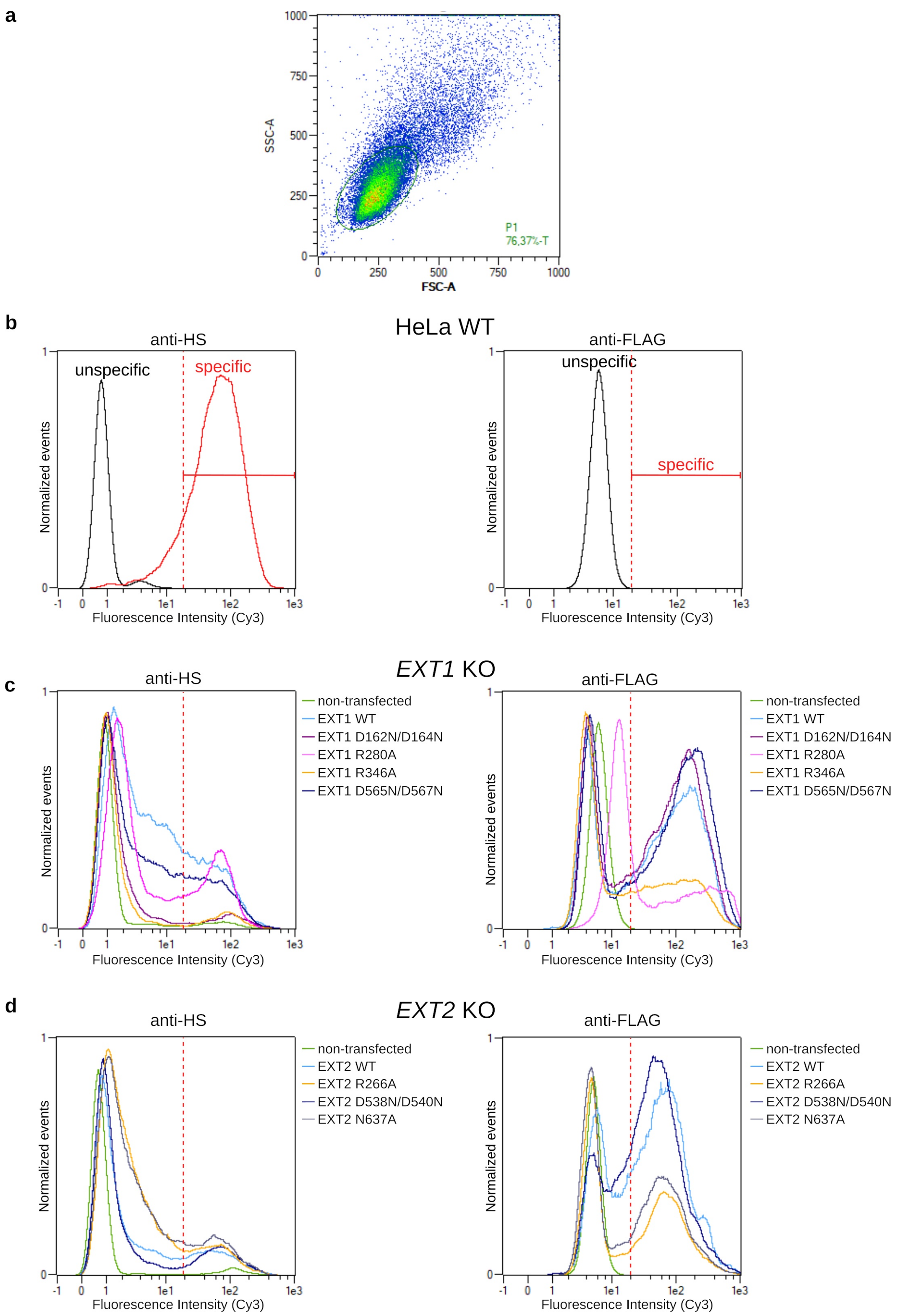

Supplementary Fig. 12 | Flow cytometry analysis of wild-type and knock-out HeLa cells. a Side versus forward scatter plot (SSC, FSC) showing the gating strategy used for analysis. **b** Flow cytometry plots from a representative experiment, in which 20 000 cells were analyzed. An arbitrary threshold (dotted red line) was

used according to negative controls (unspecific) and all events with fluorescent intensity beyond the threshold (specific) are defined as positive events (HS- or FLAG-tag specific signal). Data was analyzed applying the same threshold (dotted red line), to determine cells giving a HS- or FLAG-tag specific signal, excluding signals such as those from non transfected cells and non-specific binding of antibody. Left panel: wild-type HeLa cells were either labeled with secondary Cy3-conjugated antibody only (unspecific) or with anti-HS and secondary Cy3-conjugated antibody (specific). Right panel: wild-type HeLa cells, not carrying a FLAG-tag, were labeled with anti-FLAG and secondary Cy3-conjugated antibody (unspecific). **c** Ext1 knock-out HeLa cells (*EXT1* KO) were transfected with plasmids encoding wild-type or mutant versions of FLAG-tagged EXT1. Surface HS levels and amount of expressed EXT1 protein were quantified as described in (a). **d** Ext2 KO cell lines were transfected with wild-type or mutant versions of FLAG-tagged EXT2 and HS and EXT2 expression levels were quantified.

Supplementary Tables

Supplementary Table 1: MS-based quantitative characterization of purified human EXT1-EXT2

Accession	Protein name	Gene name	MW (Da)	Coverage (%)	Identified peptides	Quantified peptides	iBAQ
EXT2	EXT2	EXT2	78963	80.29	113	109	3.04E+08
EXT1	EXT1	EXT1	85140	75.27	84	83	1.77E+08
L0R5A1_HUMAN	Alternative protein CSF2RB	CSF2RB	11646	7.41	1	1	1.54E+07
PPB1_HUMAN	Alkaline phosphatase, placental type	ALPP	57954	64.67	35	34	4.07E+06
B4DGK4_HUMAN	Alternative protein SYT7	SYT7	13173	6.56	1	1	2.91E+05
DUS28_HUMAN	Dual specificity phosphatase 28	DUSP28	18324	6.25	1	1	9.77E+04
S12A4_HUMAN	Solute carrier family 12 member 4	SLC12A4	120650	0.74	1	1	5.01E+04
H2B1A_HUMAN	Histone H2B type 1-A	H2BC1	14167	7.09	1	1	3.90E+04
SE6L2_HUMAN	Seizure 6-like protein 2	SEZ6L2	97560	1.21	1	1	3.87E+04
VIME_HUMAN	Vimentin	VIM	53652	7.73	3	3	3.19E+04
PPBN_HUMAN	Alkaline phosphatase, germ cell type	ALPG	57377	48.68	21	1	1.18E+04
CCNB3_HUMAN	G2/mitotic-specific cyclin-B3	CCNB3	157916	0.93	1	1	1.14E+04

Supplementary Table 2: EM data collection and structure refinement statistics

**Data collection and processing**	
Microscope	Titan Krios
Voltage (kV)	300
Camera	Gatan K3-Summit
Energy Filter	Gatan Quantum-LS
Magnification	105’000x
Pixel size (Å)	0.42
Defocus range (µm)	-3.0 to -1.0
Electron exposure (e^-/Å^{-2})	46
Number of good micrographs	5’828
Initial number of particles	2’703’248
Final number of particles	286’390
Symmetry imposed	No
Map resolution (Å)	2.8
FSC threshold	0.143
Map resolution range (Å)	2.5-4.5
**Coordinate and B-factor refinement**	
Model resolution (Å)	2.56
FSC threshold	0.143
Model resolution range (Å)	2.56-3.20
Map sharpening B-factor (Å^2)	-30
Number of protein atoms (non-H)	9’261
Protein residues	1’146
Number of ligand atoms (non-H)	78
Mean B-factor protein atoms (Å^2)	71
Mean B-factor of non-protein atoms (Å^2)	78
RMSD bonds (Å)	0.003
RMSD bond angles (°)	0.58
Map CC (whole map)	0.72
Map CC (around atoms)	0.74
**Ramachandran plot**	
Favored (%)	91.4
Allowed (%)	8.4
Disallowed (%)	0.2
**Validation**	
Molprobity score	2.14
All-atom clashscore	13
Rotamer outliers (%)	0.98

Supplementary Table 3: Summary of known miss-sense mutations in EXT1 and EXT2. Literature was searched for previously described patient mutations which caused hereditary multiple exostoses and which were caused by single amino acid substitutions.

Protein	Amino Position	Mutation	References
EXT1	67	P→H	4
EXT1	164	D→H	5
EXT1	220	S→N	4
EXT1	271	Y→C	6
EXT1	280	R→ S	7
EXT1	280	R→G	7
EXT1	335	L→R	8
EXT1	339	G→D	9
EXT1	339	G→V	10
EXT1	340	R→H	4,6,7,10,11
EXT1	340	R→C	10,12
EXT1	340	R→S	13
EXT1	340	R→L	14
EXT1	341	R→G	15
EXT1	346	R→G	6
EXT1	356	V→C	11
EXT1	490	L→R	11
EXT1	644	N→Y	4
EXT1	739	G→T	15
EXT2	85	C→R	16
EXT2	128	R→W	4
EXT2	152	L→R	17
EXT2	180	R→T	12
EXT2	202	A→V	18
EXT2	223	R→P	19
EXT2	227	D→N	12
EXT2	339	C→Y	4
EXT2	380	I→T	20
EXT2	576	E→K	20

Supplementary Table 4: Primers used for site-directed mutagenesis PCR.

Mutation	Forward primer (5'- 3' sequence)	Reverse primer (5'- 3' sequence)
EXT1 D162N/D164N	ACT TTA **A**AC AGA **A**AC CAG TTG TCA CCT CAG TAT GTG CAC	CTG GT**T** TCT GT**T** TAA AGT ATC CAG ACT CAG GAC AAA GAG G
EXT1 R280A	GAC ACC **GC**G AAT GCC TTA TAT CAC GTC CAT AAC GG	C ATT C**GC** GGT GTC TGA TCC TAT CCC TGT CAG GTA C
EXT1 R346A	TCC TTC **GC**A TTC CTG GAG GCT TTG CAG GCT GCC	CAG GAA T**GC** GAA GGA CCC AAG CCT GCG ACC ACG
EXT1 D565N/D567N	GCC TT **A**AC GAG **A**AC A CG GTG CTT TCA ACA ACA GAG GTG GA	CGT GT**T** CTC GT**T** AAG GC TGA GCA CGG CGT CTG TGA TGA T
EXT2 R266A	CT GAG TAC **GC**A GAG GAC CTA GAA GCC CTC CAG GTC	CCT CT**G C**GT ACT CAG GAT GGA GAC CCA CCT GAG ATG
EXT2 D538N/D540N	GCC ATT **A**AT GAT **A**AT ATC AT TAT GCT GAC CTC TGA CGA GC	ATG AT AT**T** ATC AT**T** A ATG GC CAG AAC AGC TTC TGT CTC GAT
EXT2 N637A	G GCC **GC**C GTC ACG GGA AAA GCA GTT ATC AAG G	T GAC G**GC** GGC CAC CAG GAA GTT CAT GGC AAT ATC

Supplementary References

1. Varadi, M. *et al.* AlphaFold Protein Structure Database: massively expanding the structural coverage of protein-sequence space with high-accuracy models. *Nucleic Acids Res.* **50**, D439–D444 (2022).
2. Mirdita, M. *et al.* ColabFold: making protein folding accessible to all. *Nat. Methods* **19**, 679–682 (2022).
3. Wilson, L. F. L. *et al.* The structure of EXTL3 helps to explain the different roles of bi-domain exostosins in heparan sulfate synthesis. *Nat. Commun.* **13**, 3314 (2022).
4. Li, Y., Wang, J., Wang, Z., Tang, J. & Yu, T. A genotype-phenotype study of hereditary multiple exostoses in forty-six Chinese patients. *BMC Med. Genet.* **18**, 126 (2017).
5. Bovée, J. V. *et al.* EXT-mutation analysis and loss of heterozygosity in sporadic and hereditary osteochondromas and secondary chondrosarcomas. *Am. J. Hum. Genet.* **65**, 689–698 (1999).
6. Jamsheer, A. *et al.* Mutational screening of EXT1 and EXT2 genes in Polish patients with hereditary multiple exostoses. *J. Appl. Genet.* **55**, 183–188 (2014).
7. Raskind, W. H. *et al.* Evaluation of locus heterogeneity and EXT1 mutations in 34 families with hereditary multiple exostoses. *Hum. Mutat.* **11**, 231–239 (1998).
8. Pei, Y. *et al.* Novel mutations of EXT1 and EXT2 genes among families and sporadic cases with multiple exostoses. *Genet. Test. Mol. Biomark.* **14**, 865–872 (2010).
9. Philippe, C. *et al.* Mutation screening of the EXT1 and EXT2 genes in patients with hereditary multiple exostoses. *Am. J. Hum. Genet.* **61**, 520–528 (1997).
10. Pedrini, E. *et al.* Novel EXT1 and EXT2 mutations identified by DHPLC in Italian patients with multiple osteochondromas. *Hum. Mutat.* **26**, 280 (2005).
11. Mohaidat, Z. *et al.* Hereditary multiple osteochondromas in Jordanian patients: Mutational and immunohistochemical analysis of EXT1 and EXT2 genes. *Oncol. Lett.* **21**, 151 (2021).
12. Francannet, C. *et al.* Genotype-phenotype correlation in hereditary multiple exostoses. *J. Med. Genet.* **38**, 430–434 (2001).
13. Wuyts, W. *et al.* Identification and characterization of a novel member of the EXT gene family, EXTL2. *Eur. J. Hum. Genet. EJHG* **5**, 382–389 (1997).
14. Hecht, J. T. *et al.* Hereditary multiple exostoses (EXT): mutational studies of familial EXT1 cases and EXT-associated malignancies. *Am. J. Hum. Genet.* **60**, 80–86 (1997).
15. Al-Zayed, Z. *et al.* Mutation spectrum of EXT1 and EXT2 in the Saudi patients with hereditary multiple exostoses. *Orphanet J. Rare Dis.* **16**, 100 (2021).
16. Park, K. J. *et al.* Germline mutations in the EXT1 and EXT2 genes in Korean patients with hereditary multiple exostoses. *J. Hum. Genet.* **44**, 230–234 (1999).
17. Xu, L. *et al.* Mutation analysis of hereditary multiple exostoses in the Chinese. *Hum. Genet.* **105**, 45–50 (1999).
18. Seki, H. *et al.* Mutation frequencies of EXT1 and EXT2 in 43 Japanese families with hereditary multiple exostoses. *Am. J. Med. Genet.* **99**, 59–62 (2001).
19. Shi, Y. R., Wu, J. Y., Tsai, F. J., Lee, C. C. & Tsai, C. H. An R223P mutation in EXT2 gene causes hereditary multiple exostoses. *Hum. Mutat.* **15**, 390–391 (2000).
20. Gigante, M. *et al.* Ext-mutation analysis in Italian sporadic and hereditary osteochondromas. *Int. J. Cancer* **95**, 378–383 (2001).
