# Supplementary material for: Structure of the human heparan sulfate polymerase complex EXT1-EXT2
Source: Nat Commun. 2022 Nov 19;13:7110. doi: 10.1038/s41467-022-34882-6 (PMC9675754; doi:10.1038/s41467-022-34882-6)
Supplement: Supplementary file 4 — Source data [file 41467_2022_34882_MOESM4_ESM.zip › Source_data/Source Data Summary.pdf]

Description of Additional Supplementary Files

File name: Source Data 1
Description: Uncropped SDS-PAGE gel of Figure 1a showing purification of EXT1-EXT2 complex. (.png)

File name: Source Data 2
Description: Mass photometry chromatogram data of purified EXT1-EXT2 complex presented in Figure 1b. (.xlsx)

File name: Source Data 3
Description: Uncropped FACE gel of Figure 1c showing the *in vitro* N-acetylglucosamine and glucuronic acid transfer activities of EXT1-EXT2 complex. (.png)

File name: Source Data 4
Description: Uncropped FACE gel of Figure 3f displaying the metal dependency of the *in vitro* N-acetylglucosamine and glucuronic acid transfer activities of the EXT1-EXT2 complex. (.png)

File name: Source Data 5
Description: Uncropped FACE gel of Figure 4b showing the *in vitro* N-acetylglucosamine and glucuronic acid transfer activities of mutant EXT1-EXT2 complexes. (.png)

File name: Source Data 6
Description: Cell surface heparan sulfate levels (anti-10E4) and flag-tagged EXT2 protein levels (anti-FLAG) quantified by FACS analysis presented in Figure 4c. (.xlsx)

File name: Source Data 7
Description: Uncropped FACE gel of Figure 5b showing a time course of *in vitro* chain elongation catalyzed by the EXT1-EXT2 complex. (.png)

File name: Source Data 8
Description: Uncropped SDS-PAGE gel of Figure 11a showing purification of mutant EXT1-EXT2 complexes. (.png)

File name: Source Data 9
Description: Nano differential scanning fluorimetry data shown in Supplementary Fig. 11c that was used to determine the melting temperatures of EXT1-EXT2 wild-type and mutant complexes. (.xlsx)
