# Supplementary figures and images for: Structure of the human heparan sulfate polymerase complex EXT1-EXT2
Source: Nat Commun. 2022 Nov 19;13:7110. doi: 10.1038/s41467-022-34882-6 (PMC9675754; doi:10.1038/s41467-022-34882-6)

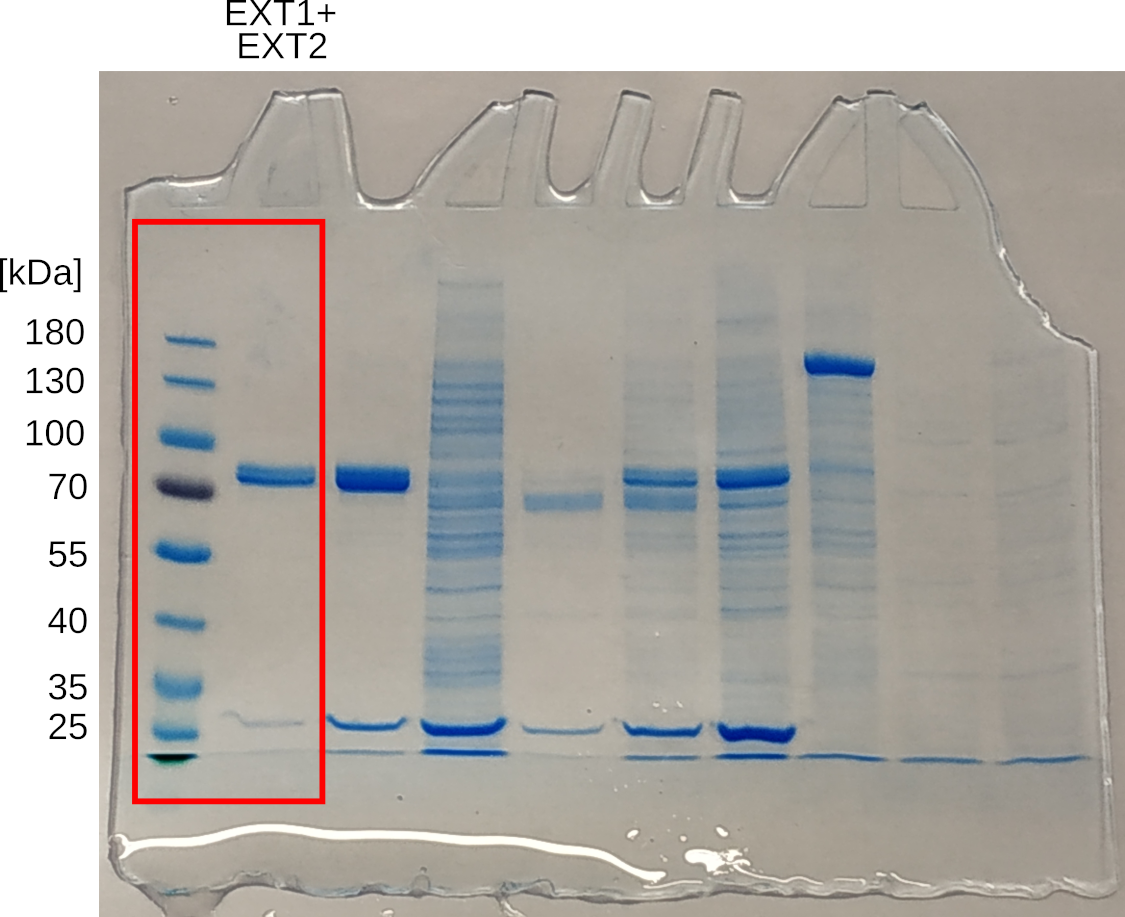

Supplement: Supplementary file 4 — Source data [file 41467_2022_34882_MOESM4_ESM.zip › Source_data/Source Data 1.png]

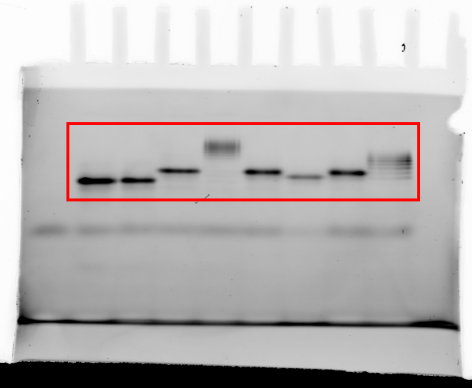

Supplement: Supplementary file 4 — Source data [file 41467_2022_34882_MOESM4_ESM.zip › Source_data/Source Data 3.png]

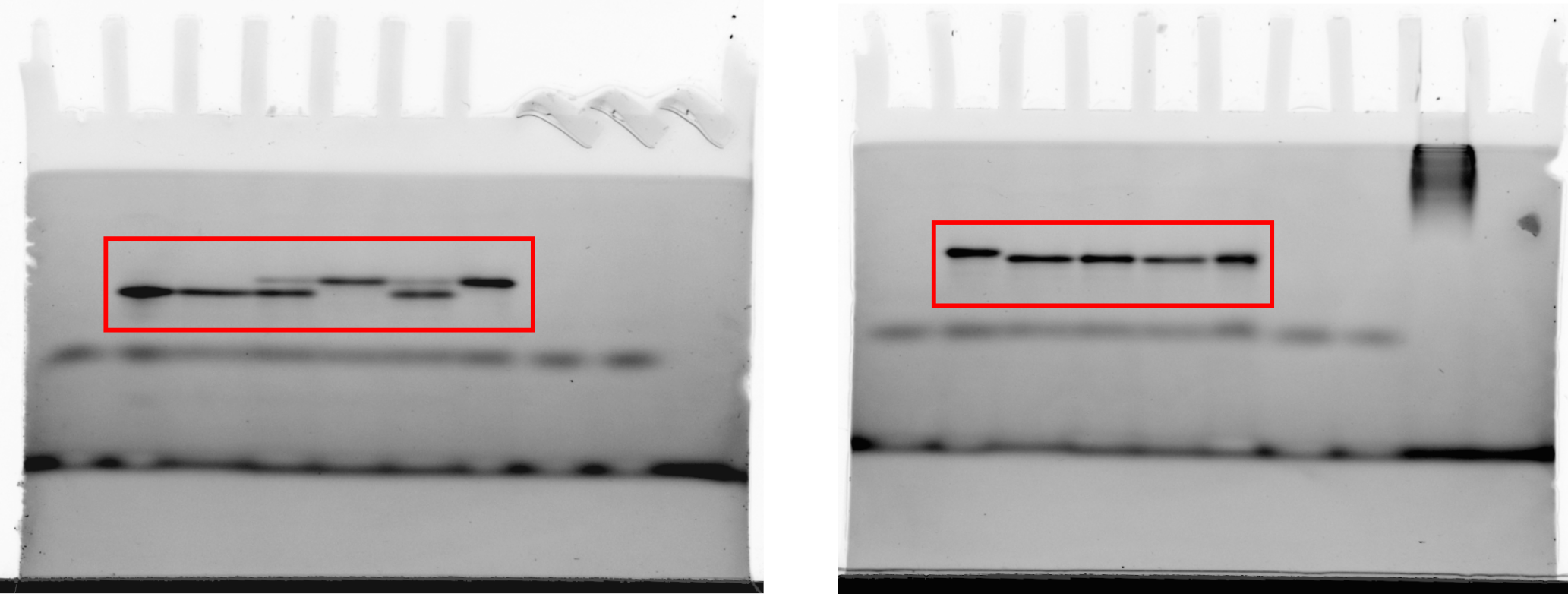

Supplement: Supplementary file 4 — Source data [file 41467_2022_34882_MOESM4_ESM.zip › Source_data/Source Data 4.png]

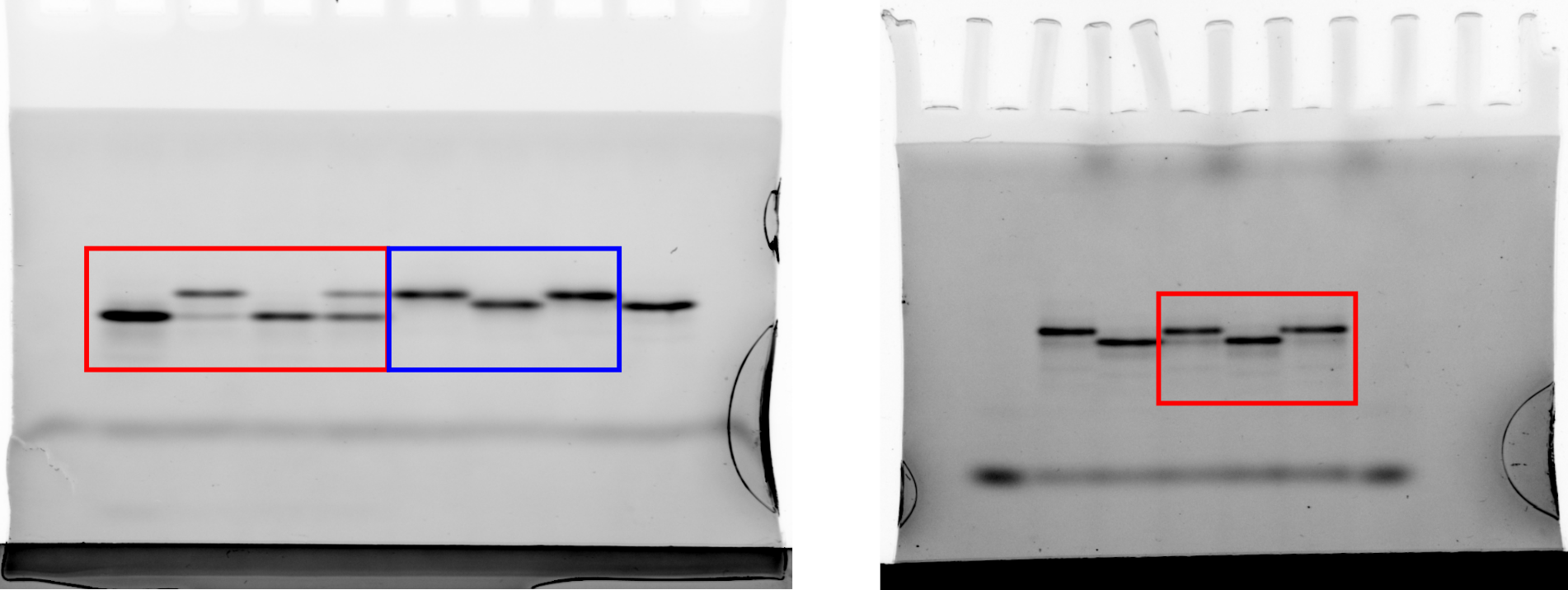

Supplement: Supplementary file 4 — Source data [file 41467_2022_34882_MOESM4_ESM.zip › Source_data/Source Data 5.png]

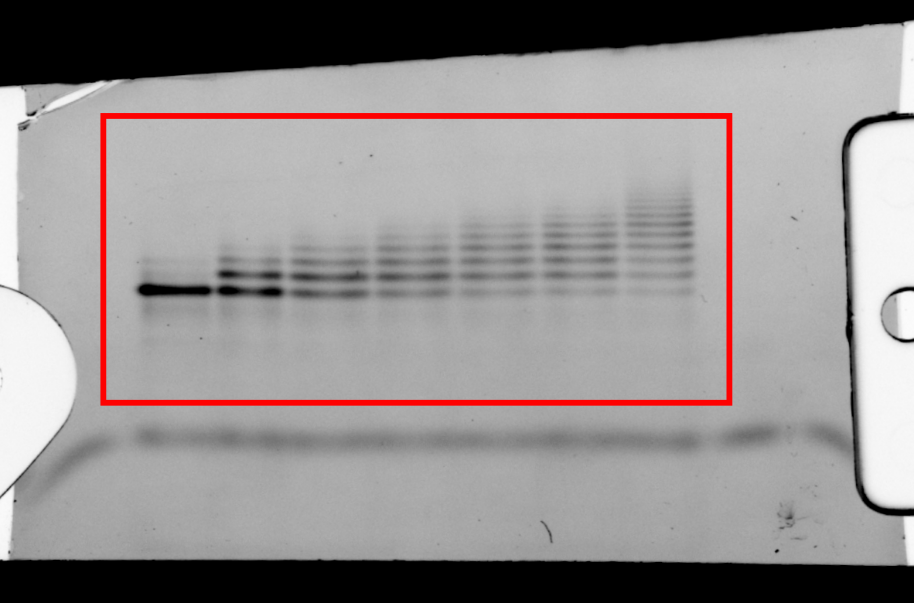

Supplement: Supplementary file 4 — Source data [file 41467_2022_34882_MOESM4_ESM.zip › Source_data/Source Data 7.png]

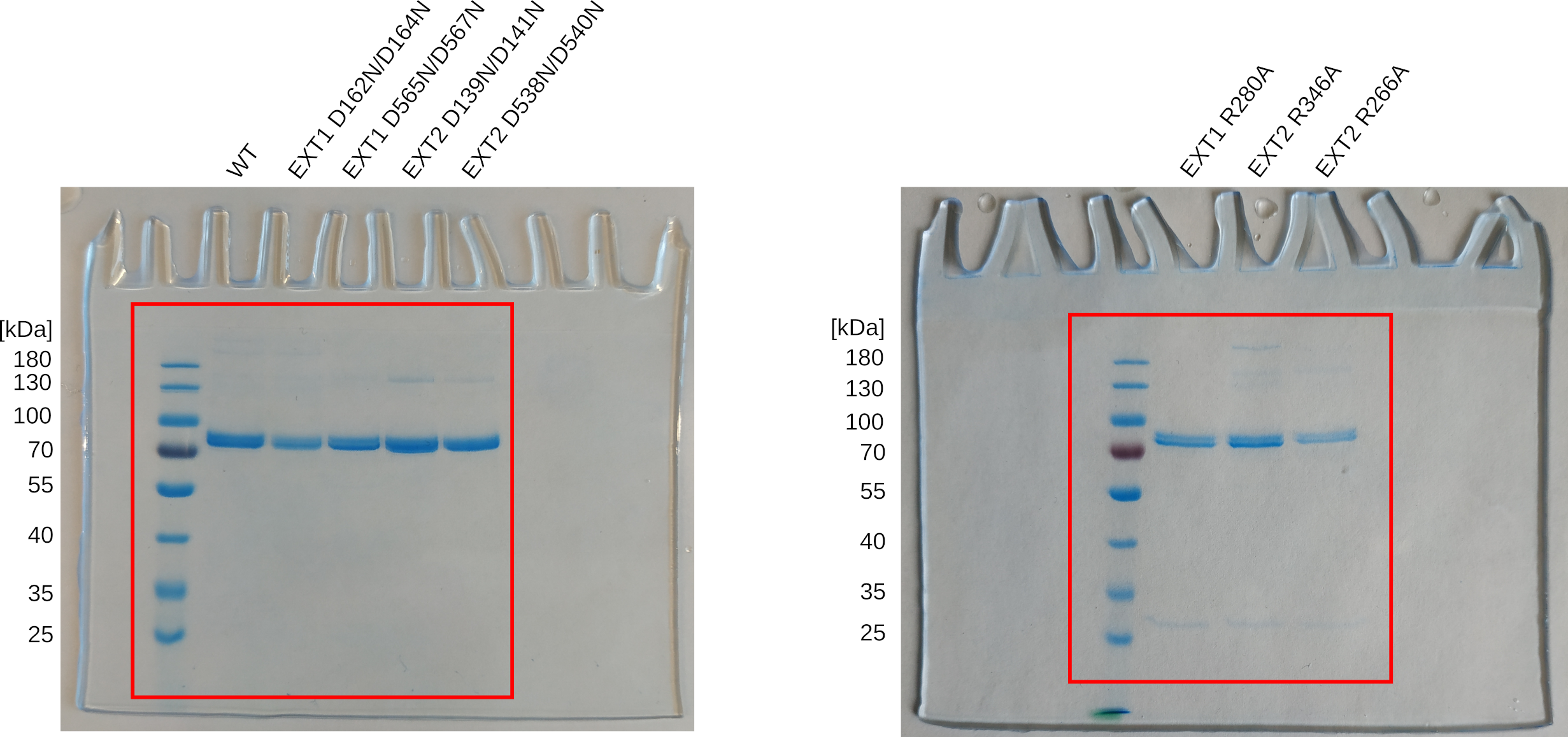

Supplement: Supplementary file 4 — Source data [file 41467_2022_34882_MOESM4_ESM.zip › Source_data/Source Data 8.png]
